# A New Clinical Thermometer

**Published:** 1908-04-04

**Authors:** 


### NEW APPLIANCES AND THINGS MEDICAL.

[We shall be glad to receive, at our Office, 28 & 29 Southampton Street, Strand, London, W.C., from the manufacturers, specimens of all new preparations and appliances which may be brought out from time to time.]

### A NEW CLINICAL THERMOMETER.

Dr. L. Usher Somers, of West Bromwich, has forwarded us a specimen of a new clinical thermometer made according to his designs by Messrs. Southall Bros. and Barclay, Ltd., Birmingham. The instrument, which is similar in size and general make to the ordinary half-minute clinical thermometers, differs from the more familiar types in the system of graduation that has been adopted. In the Usher-Somers thermometer the zero corresponds to the normal physiological temperature average (98.4° F.) and the graduation extends above this point to +10 and below it to −5. Reduction of this scale to Fahrenheit is easy enough so long as the essential point is remembered that the zero corresponds to 98.4° F. Thus it is only necessary to add or to subtract a degree according to whether the reading is plus or minus. Special charts are, however, supplied with the instrument which facilitate the reduction, and for those who prefer centigrade readings, special instruments are made in which the zero corresponds to the centigrade normal. We have given the instrument supplied to us a careful trial and have found it accurate and convenient. The bold outlines of the scale, which for the sake of clearness are filled in in red, make it extremely easy to read, and we venture to think that it will prove a popular type. The price is not stated, but particulars may be had on application to the makers.

28 & 29 SOUTHAMPTON STREET, STRAND, LONDON, W.C.

